# New Perspectives for Postmortem Human Satellite Cells of Different Embryological Origin

**DOI:** 10.3389/fphys.2022.886149

**Published:** 2022-05-25

**Authors:** Tiziana Pietrangelo, Roberto Demontis, Carmen Santangelo, Niccolò Pini, Martina Bonelli, Enrica Rosato, Paola Roberti, Marcello Locatelli, Angela Tartaglia, Lorenzo Marramiero, Vittore Verratti, Danilo Bondi, Stefania Fulle, Ernesto D’Aloja, Cristian D’Ovidio

**Affiliations:** ^1^ Laboratory of Functional Evaluation and Cellular Physiology, Department Neuroscience Imaging and Clinical Sciences, University “G. d’Annunzio” of Chieti-Pescara, Chieti, Italy; ^2^ Interuniversity Institute of Myology (IIM), Chieti, Italy; ^3^ Department of Medical Sciences and Public Health, Section of Legal Medicine, University of Cagliari, Cagliari, Italy; ^4^ Department of Medicine and Aging Sciences, Section of Legal Medicine, University “G. d’Annunzio” of Chieti-Pescara, Chieti, Italy; ^5^ Department of Pharmacy, University “G. d’Annunzio” of Chieti-Pescara, Chieti, Italy; ^6^ Department of Psychological, Health and Territorial Sciences, University “G. d’Annunzio” of Chieti-Pescara, Chieti, Italy

**Keywords:** satellite cells, skeletal muscle regeneration, postmortem, presomitic muscles, somitic muscles, embryonic origin, organoids

## Abstract

Human postmortem skeletal muscles are a unique source of satellite cells for skeletal muscle regenerative studies. Presomite and somite satellite cells obtained by postmortem muscles have been established as populations of human skeletal muscle precursor cells able to proliferate and differentiate *in vitro*. It is extremely interesting to have access to a large amount of postmortem human skeletal muscle precursor cells, especially from craniofacial as well as limb skeletal muscles in order to evaluate their potential application not only for the fundamental understanding of muscle physiology and diseases but also for drug testing in a challenging 3D-shaping muscles like skeletal muscle microphysiological systems.

## Introduction

The skeletal muscle regeneration is a fundamental biological aspect based on activation of adult stem cells and satellite cells (SCs) that accompany humans throughout their entire life. Indeed, SCs have been found alive several days *post mortem*, and these cells are able to generate a progeny through few steps *in vivo* (Latil et al., 2012; Scott et al., 2013). However, scarce data exist on postmortem SCs and any of their functional features. Not to mention the craniofacial muscle stem cell biology, which only recently has started to be gathered (Cheng et al., 2021). Indeed, most data of these muscles remain at the level of tissue description, few indications about their SCs, and no data exist at the postmortem level for presomite SC features. Briefly, it is worth remembering that the skeletal muscles originate from two distinct embryonic districts: craniofacial muscles like thyrohyoid derived from pharyngeal arches. The striated muscle of each arch (sometimes termed branchiomeric) is derived from the rostral continuation of the paraxial mesoderm. The paraxial mesoderm of the head rostral to the occipital region is unsegmented. The somitomeres, which are spherical clusters of mesenchymal cells in the presomitic mesoderm, presage the segmentation of somites in the paraxial mesoderm. The trunk/limb muscles derive from the lateral plate mesoderm. In this perspective, the human postmortem SC functions, both from trunk and craniofacial derivations, deserve attention and need to be explored to understand their regenerative potential and possible applications (Feige et al., 2018).

We aimed to investigate some features of postmortem *in vitro* SCs, namely human muscle precursor cells (hMPCs), both obtained from somite and pharyngeal arch muscles, specifically the retainment of alive postmortem hMPCs and their ability to proliferate and differentiate along with their intracellular calcium signaling, which was never investigated before, to the best of our knowledge.

## Materials and Methods

The presomite muscles (from pharyngeal arches) thyrohyoid muscles was obtained from the corpses of 40, 43, 45, and 71 years old, and the somite muscles considered were *ileopsoas* obtained from the same corpses. Informed consents were signed by the deceased subjects’ family members according to the Ethic Committee approval (COET n 6065-04.03.2021).

Histopathological examination of tissue samples obtained during autopsies showed no signs of pathologies that could invalidate the value of further investigations. The muscle has been sampled and immediately immersed in sterile solution containing HAM’s F10 and gentamicin. Each sampling involved the removal of tissue fragments for each muscle through a small accessory cutaneous cut. The sampling on the craniofacial muscle was carried out on the lateral margin, 2 cm from the clavicular insertion; the removal of the vastus lateral muscle will instead be carried out on the lateral margin 2 cm from its origin at the level of the greater trochanter. The weight of the muscle was about 1 g each.

Two different protocols were used to collect the muscle samples: in the first protocol, the muscle sample was put into a physiological medium containing HAM’s F10 and gentamicin and stored for 24 h at 4°C. The medium was renewed three times in order to wash the blood residues, and then the muscle sample was treated for explant formation. In the second protocol, the muscle sample was stored in liquid nitrogen in fetal bovine serum (FBS, Euroclone) + 10% of dimethyl sulfoxide (DMSO, Sigma-Aldrich). Frozen dissected muscle biopsies were thawed at 37°C and washed with PBS before the treatment for explant formation.

### Satellite Cell Cultures

Satellite cells were isolated from muscle tissues using the explant procedure as previously described (Pietrangelo et al., 2011 and Pietrangelo et al., 2015). Briefly, small pieces of muscles, named explants, obtained by mincing using sterilized scissors, were put on Petri dishes with a drop of FBS and stored in an incubator at 37°C, 5% CO_2_, and saturated humidity. During the following 2 weeks, the SCs migrated out of the explants and started to proliferate. To be coherent with the literature, we name these cells hMPCs. After detaching with trypsin-EDTA, the cells were counted, and the population doubling level was calculated at each passage with the following equation: log_10_(N/n)/ln_2_ with N as the number of cells at the time of the passage and n as the number of cells initially plated. At the first passage, the cell population was considered at 1 population doubling level (PDL). The proliferative state was maintained by feeding the hMPCs with a growth medium (GM) containing (% vol/vol): HAM’s F10 (Euroclone), 0.1 gentamycin and 1 penicillin/streptomycin 100X (Euroclone), 20 FBS heat-inactivated (56°C, 36 min) (Hyclone), and 1 l-Glutamax 100 × (Gibco). The percentages of myogenic cells were obtained using an immunocytochemistry assay for the marker desmin and with biotinylated streptavidin-AP kits (LSAB + System-AP Universal kits; Cat. No. K0678; DAKO, DakoCytomation, Glostrup, Denmark). Cell cultures with desmin positivity of less than 70% were sorted for surface myogenic markers using CD56 (mouse monoclonal antibody (Abcam, Cambridge, UK)) by flow cytometric analysis (Di Filippo et al., 2016) in order to achieve high myogenic populations.

The differentiation was induced by feeding the hMPCs with a differentiation medium (DM) containing (% vol/vol): DMEM high glucose, 0.1 gentamycin, five heat-inactivated HS (56°C, 36 min), 10 μg/ml insulin, 100 μg/ml apo-tranferrin (Sigma), 1 sodium pyruvate 100 mM, 1 penicillin/streptomycin 100 ×, and 1 l- glutamine 100 × for 7 and 12 days. The myotubes were positive for both the primary antibody against desmin and myosin heavy chain using the MF20 anti-MHC monoclonal antibody (diluted 1:50; Developmental Studies Hybridoma Bank, University of Iowa, Iowa City, IA, United States).

### Intracellular Calcium Concentration Measurements

The proliferating hMPCs in the range of 2–3 PDL and differentiated myotubes (7 and 12 days differentiation) were loaded with Fura2-AM (final concentration, 5 μM) for 30 min, washed by removal solution, and incubated for further 30 min at 37°C prior to the intracellular calcium concentration at the cytosolic level ([Ca^2+^]_i_) measurement, to allow intracellular Fura2-AM de-esterification. The experiments were performed at the room temperature of 21°C and sea-level O_2_ and CO_2_ partial pressure. Images were acquired using the procedures and set-up described by Pietrangelo et al. (2015).

### Data Analysis

The analyses, mean and standard deviation, and unpaired t-tests were performed using GraphPad Prism Software, version 5 (GraphPad Software, La Jolla, United States).

## Results

We isolated postmortem hMPCs from thyrohyoid and ileopsoas. Their desmin positivity was tested, and if it was less than 70%, the cells were sorted for CD56 marker. The cell yield was 5 × 10^3^ ± 700 per mg of presomite muscles and for somite muscles, was 5 × 10^3^ ± 970 cells. Similar results were obtained for the postmortem hMPCs from samples derived by the two procedures described before in Materials and Methods, specifically samples stored at 4°C for 24 h and samples frozen for 2 weeks in liquid nitrogen.

**FIGURE 1:**
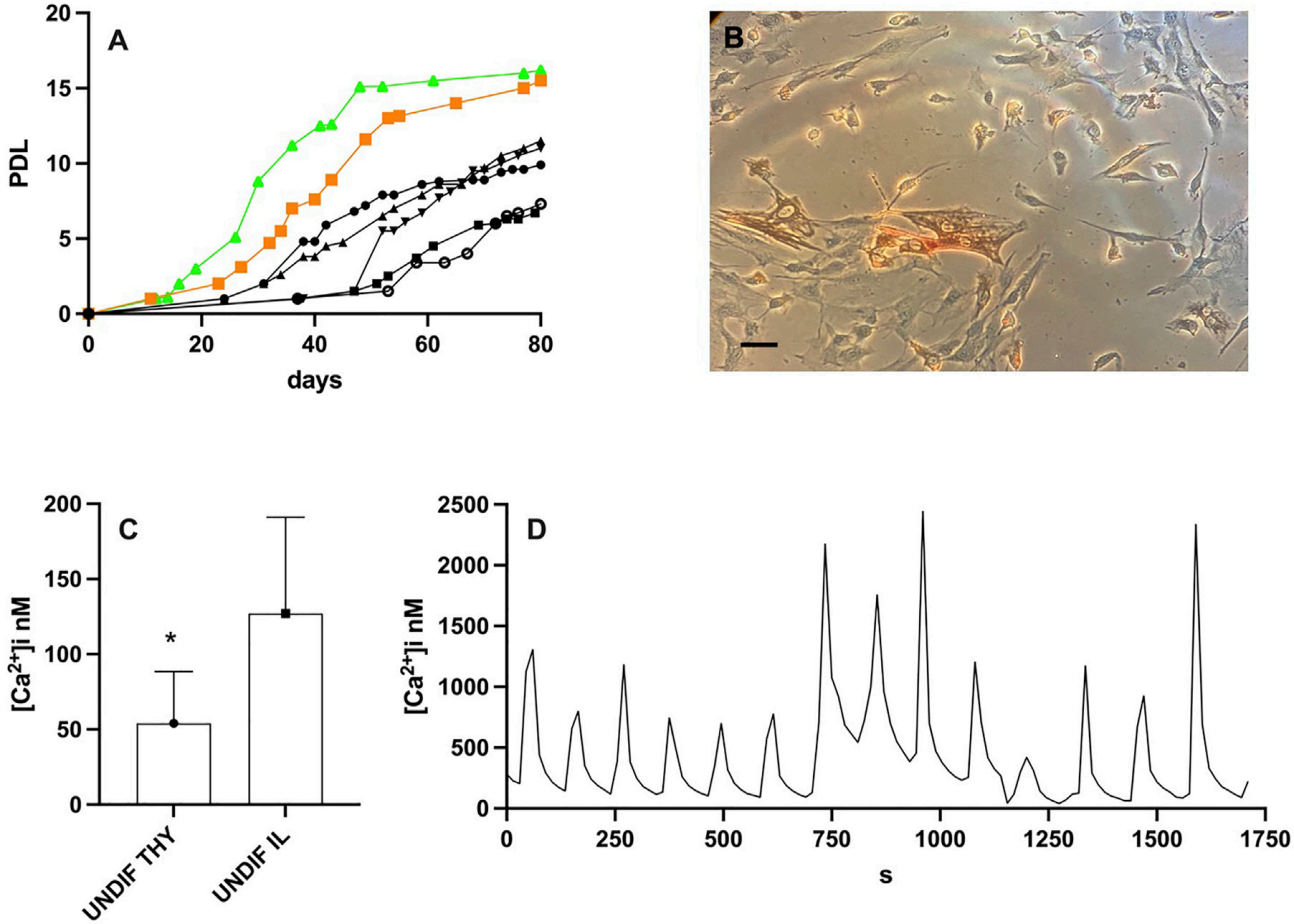
Results on undifferentiated thyrohyoid and ileopsoas hMPCs. **(A)** Population doubling level (PDL) of postmortem hMPCs derived from thyrohyoid muscle obtained from 40– (orange squared symbol) and from iliopsoas muscle obtained from 71– (green triangle symbols) year-old corpses while black symbols represent the PDL of five different hMPC populations derived from vastus lateralis of alive donors in the range of 40–71 years old. **(B)** Picture of thyrohyoid hMPCs in Petri dishes. **(C)** The graph shows mean and standard deviation of [Ca^2+^]_i_ measurement on undifferentiated thyrohyoid (UNDIF THY) and ileopsoas (UNDIF IL) hMPCs. They significantly differ with *p* ≤ 0.05. **(D)** Representative [Ca^2+^]_i_ oscillation recorded on thyrohyoid hMPCs. The bar in panel B represents 100 μm.

Previously, we have demonstrated that the hMPCs obtained from alive donors, migrated out of the explants within 1 month and reached the proliferative senescence (cells do not duplicate anymore) in about 3–4 months (Pietrangelo et al., 2009). Postmortem hMPCs, both from presomite and somite muscles, migrated out of the explant within 15 days, faster than those migrating out of the explants from muscles of living volunteers. Postmortem hMPCs showed an increased proliferation rate (Figure 1A, postmortem hMPCs in green and orange curves) compared with hMPCs derived from alive donors (Figure 1A black curves). Moreover, postmortem thyrohyoid hMPCs (Figure 1B) proliferate faster than postmortem ileopsoas hMPCs (green vs. orange curve in Figure 1).

We measured the [Ca^2+^]_i_ in postmortem hMPCs as proliferating undifferentiated cells under resting conditions. The [Ca^2+^]_i_ in thyrohyoid hMPCs was significantly less than [Ca^2+^]_i_ in ileopsoas hMPCs (Figure 1C). Moreover, thyrohyoid hMPCs showed peculiar spontaneous intracellular [Ca^2+^] waves. The [Ca^2+^]_i_ regularly oscillates with a peak followed by a resting level for a period of few minutes. The [Ca^2+^]_i_ oscillation was recorded for 50 ± 15 min. These oscillatory pathways showed different frequencies. Figure 1D shows a representative trace with a frequency of about 30 [Ca^2+^]_i_ peaks per hour; we also recorded slower frequencies of about 13 and 6 [Ca^2+^]_i_ peaks per hour. We never recorded oscillatory [Ca^2+^]_i_ in ileopsoas postmortem hMPCs.

**FIGURE 2:**
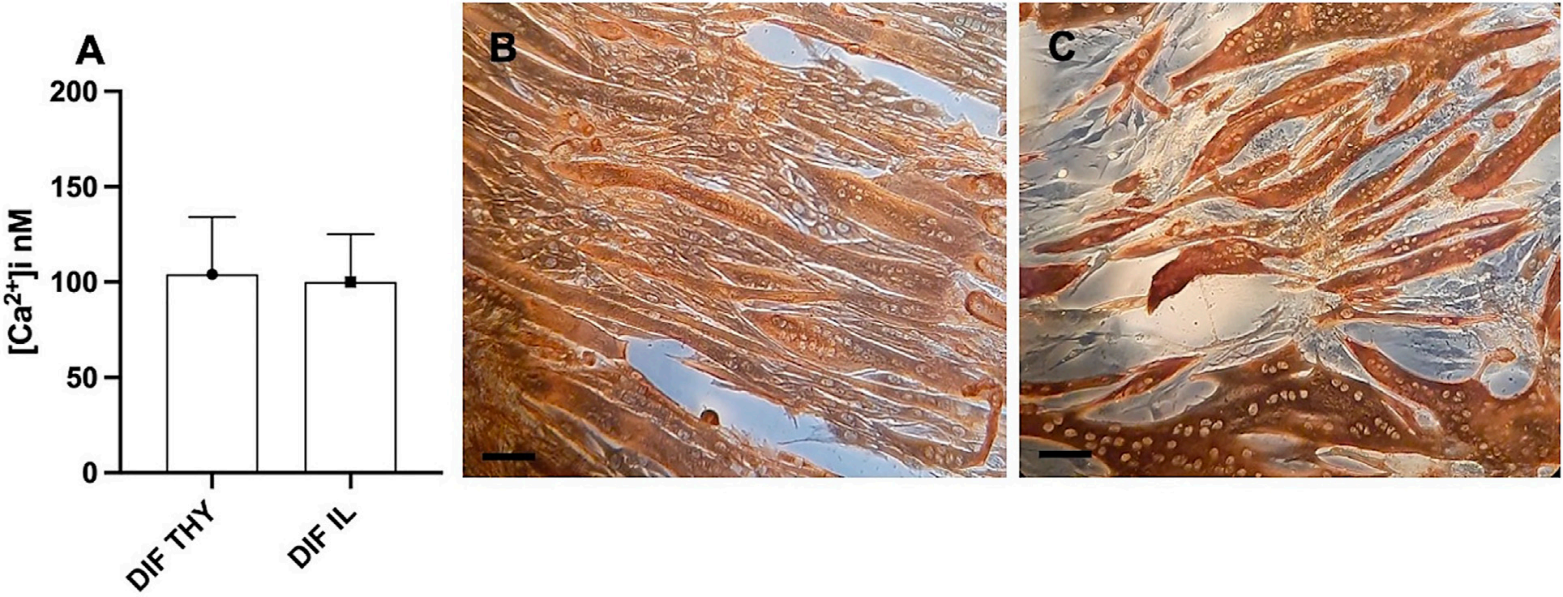
Results of differentiated postmortem hMPCs. **(A)** [Ca^2+^]_i_ recorded as the basal level in differentiated hMPCs derived from both thyrohyoid (DIF TY) and ileopsoas (DIF IL) muscles. **(B,C)** Representative images of immunostaining for desmin **(B)** and myosin heavy chain proteins **(C)** on myotubes derived from postmortem thyrohyoid hMPCs. The bars represent 100 μm.

We analyzed the differentiation process in both somite and presomite hMPCs along with their [Ca^2+^]_i_. Figure 2B shows multinucleated myotubes of thyrohyoid hMPCs stained for desmin, while panel C stained for myosin heavy chain protein expression. It can be observed that not all the hMPCs were fused into myotubes at 7 days despite being myogenic cells (panel C). Prolonging the differentiation at 10–12 days, about 90% of hMPCs formed myotubes. We measured the [Ca^2+^]_i_ in myotubes (panel D) under resting conditions (Figure 2A) and found similar [Ca^2+^]_i_ in presomite and somite myotubes. We stimulated the myotubes with 500 μM nicotine in order to reveal the presence and the opening of achetylcholine channels. The somite-differentiated hMPCs showed responsiveness to nicotine at 7 days while presomite myotubes showed later at 10–12 days of differentiation.

## Discussion and Conclusion

To our current understanding, no one has previously established that postmortem presomite muscles were able to release hMPCs *in vitro* to be cultured and differentiated. Our results demonstrated that postmortem thyrohyoid hMPCs proliferate with features similar to somite ones, and both lineages are able to come out of the explants earlier and proliferate faster than those from biopsies obtained from alive donors. Postmortem thyrohyoid hMPCs showed peculiar spontaneous [Ca^2+^] waves lasting tens of minutes. It is worth mentioning that embryological pharyngeal arches originate in not only neck muscles but also cardiomyocytes, which have been demonstrated to be able to give the oscillatory pattern of resting cytosolic calcium (Eisner et al., 2017; Cheng et al., 2021). This creates proactive behavior of the presomite hMPCs, a sort of pacemaking prone to contraction. Considering the large amount of calcium mobilization during oscillatory waves (peak more than 1 μM), we thought that this pattern could be a death signal, but it is worth mentioning that any significant apoptotic cell increase has been recorded on thyroyoid hMPCs.

The [Ca^2+^]_i_ under resting conditions was significantly less in presomite hMPCs with respect to somite and differentiated myotubes. Following these results, the human postmortem hMPCs functions, both from somite and craniofacial muscles, need to be further explored to deeply understand their regenerative potential and possible applications.

Distinct lineages of SCs are responsible for both head and trunk/limb muscle tissue formation with specific genetic differences in fiber development (Harel et al., 2009; Tzahor, 2009). It has been demonstrated that mutant mice lines that show no development of trunk and limb muscles are still able to form embryonic and fetal muscles of the head (Rodriguez-Outeiriño et al., 2021; Rudnicki et al., 1993).

Scott et al. (2013) well described the isolation of human skeletal muscle satellite cells from postmortem somitic muscles and proposed their utility. Recently, Feige et al. (2021) established a “novel model” to evaluate the human satellite cell fate using postmortem intact human muscle myofibers with muscle stem cells within the niche microenvironment but only on the somite human psoas.

However, to our knowledge, the data we reported in this study are the first demonstration that pre-somitic SCs can be achieved from postmortem presomite muscles, established in culture, and used for studies on muscle regeneration despite the fact that craniofacial muscle regeneration has physiological peculiar properties and promising characteristics observed during aging or muscle disease. Interestingly, eye extrinsic muscles do not show signs of sarcopenia and are less affected by muscular dystrophies (McLoon et al., 2007; La Rovere et al., 2014; Cheng et al., 2021). The amount of SCs we obtained by thyrohyoid is also impressive: if we consider that 1 mg of muscle furnished about 5 × 10^3^ cells at the first PDL, we had 1 g of muscle. This means achieving more than 100 × 10^6^ at the first passage *in vitro* during culture. Considering that one of the main limitations in microphysiological systems like building muscle organoids is the requirement of large number of adult stem cells (Low et al., 2021); we suggest overcoming it by taking advantage of large-scale and homogeneous postmortem hMPC populations. Human biopsy sampling on living volunteers presents some limitations as very small muscle sampling was performed to avoid the scar tissue formation/muscle function impairment, very rare or rather impossibility to sampling presomite muscle. Postmortem presomite and somite sampling basically overcomes or postpones these limitations. Indeed, the coroner has cut a large amount of muscle for investigations, and further it is worth mentioning that our preliminary data suggest that postmortem hMPCs lifespan significantly increases, even with a certain variability, with respect to the lifespan of hMPCs populations obtained by living donors. We did not test the extent of lifespan yet, but it seems present in each hMPC population we obtained. It will be very interesting to investigate if postmortem thyrohyoid hMPCs change their Hayflick limit for duplicative senescence with respect to somite ones.

Using postmortem SCs, we can produce not only a large number of cells useful for organ-on-chip and organoid differentiation protocol but also allow having a structure mimicking nerve-dependent skeletal muscle contraction. Muscle fiber contractions are visible in about 20 days and can be maintained over a long period, thanks to the production of innervated multinucleated mature skeletal muscle fibers (Mazaleyrat et al., 2020).

Since the discovery in humans of somite cell reprogramming into pluripotent stem cells (hiPSCs), several protocols of cell lineage proliferation and differentiation have been created aimed at modeling physiological programs, starting the modern stem cell-based regenerative medicine (Ortiz-Vitali and Darabi, 2019). Matsuda et al. (2020) used iPSCs for studying the stepwise origin that recapitulates more complex features of human mesoderm development and *in vitro* induction of presomite mesoderm on human somitogenesis, with the interesting demonstration of good achievement on spondylocostal dysostosis. However, the skeletal muscle differentiation studies based on iPSCs have lagged behind those of other cell lineages. The protocols for generating mature presomite muscle fibers with sarcolemmal organization using iPSCs remain unexplored, and the investigation of the complexity of mature skeletal muscle is still lacking. Not to mention modeling and investigating specific interesting features of presomite skeletal muscles under dystrophic conditions, in which presomite muscles survive longer than other muscles also under very severe conditions like Duchenne muscular dystrophies.

Overall, these efforts pave the way to demonstrate the great potential of presomite iPSCs for disease modeling considering several pathologies, as well as helping identify new pathologic mechanisms involved (Dutta et al., 2017). Considering regenerative medicine, several other conditions can take advantage of the studies on presomite 2D and 3D organoids, as those for the identification of physiological or pathological mechanisms underlying the regeneration process as well as drug interference (Ostrovidov et al., 2019).

Amazing possibilities may emerge from the advancement of our perspective. First, the definition of protocols for muscle stem cells-derived organ-on-chip and organoids;. second, the subsequent disease modeling and no-patient clinical trials (Mummery et al., 2014); and third, extending the field of regenerative medicine models for organ transplants (Hoogduijn et al., 2020) with postmortem models.

In perspective, this work opens a new field of investigation on postmortem satellite cells from presomite and somite skeletal muscle finalized to specific biobanks to be used for organoid formation.

In conclusion, our perspective work is in line with the recent advances in regenerative and precision medicine and the need of understanding the fundamental mechanisms in presomite and somite SC models and their role in microphysiological systems for skeletal muscle studies (Jalal et al., 2021). Moreover, our ability to collect and manage postmortem presomite as well as somite hMPCs can significantly contribute to creating a new biobank shareable with researchers involved in physiological and pathological studies on skeletal muscle.

## Data Availability

The raw data supporting the conclusion of this article will be made available by the authors, without undue reservation.
